# Utility of High-Frequency Ultrasound in Preoperative Evaluation of the Thickness of Cutaneous Melanoma

**DOI:** 10.3390/diagnostics16010170

**Published:** 2026-01-05

**Authors:** Yang Zhao, Feiyue Yang, Danhua Li, Qiao Wang, Lehang Guo, Weiwei Ren, Dandan Shan, Chuan Qin

**Affiliations:** 1 Department of Medical Ultrasound, Jinshan Hospital of Fudan University, Shanghai 201508, Chinadenise2786@126.com (D.L.); 2 Department of Medical Ultrasound, Shanghai Tenth People’s Hospital, School of Medicine, Tongji University, Shanghai 200072, China; 3 Shanghai Engineering Research Center of Ultrasound Diagnosis and Treatment, Shanghai 200072, China; 4 Department of Medical Ultrasound, Shanghai Skin Disease Hospital, Ultrasound Research and Education Institute, School of Medicine, Tongji University, Shanghai 200443, China

**Keywords:** cutaneous melanoma, CM, high-frequency ultrasound, HFUS, Breslow thickness

## Abstract

**Introduction**: The Breslow thickness of cutaneous melanoma (CM) is related to the surgical approach and is usually measured using a preoperative biopsy. However, tumor thickness is often underestimated by partial biopsies. **Objectives**: To identify whether high-frequency ultrasound (HFUS) can improve the accuracy of preoperative detection of Breslow thickness. **Methods**: Partial biopsies and HFUS measurements of Breslow thickness were analyzed in 17 patients with CM. Postoperative histopathologic examination is considered the gold standard. In different thicknesses, HFUS and partial biopsy were compared with postoperative pathology and their effects on tumor T staging and surgical margins. **Results**: The mean (±SD) Breslow thicknesses measured using HFUS, partial biopsy, and postoperative histopathology were 3.5 ± 2.2 mm, 1.8 ± 1.1 mm, and 3.2 ± 2.1 mm, respectively. The correlation coefficients were 0.76 (partial biopsy and postoperative histopathology) and 0.96 (HFUS and postoperative histopathology), respectively (all *p* < 0.01). Partial biopsy underestimated the Breslow thickness, and with a gradual increase in CM thickness, the underestimation became increasingly obvious. Partial biopsy led to an underestimation of T staging of the tumor in 8 (8/17 [47.1%]) patients, 4 (4/8 [50.0%]) of whom may have had insufficient surgical peripheral margins. Conversely, HFUS exhibited a significant downward trend of underestimation (0/17 [0%], *p* = 0.003). However, only 2 (2/17 [11.8%]) patients had slight overestimation for T staging (*p* = 0.485), and none of the overestimations changed the surgical margins. **Conclusions**: HFUS can provide an accurate preoperative assessment of Breslow thickness, and correlates better with postoperative histopathology.

## 1. Introduction

Cutaneous melanoma (CM) is a highly aggressive skin tumor that is responsible for 90% of skin tumor-related deaths. In 2020, there were 325,000 new cases and 57,000 deaths globally, with projections of 510,000 new cases (a 57% increase) and 96,000 deaths (a 68% increase) by 2040 [1]. Despite being one of the most lethal skin cancers, CM has a 5-year relative survival rate of over 93% with early detection and treatment [2].

The vertical tumor thickness, known as Breslow thickness, plays a crucial role in the treatment and prognosis of CM [3]. This measurement is determined on histological samples using an optical micrometer, representing the depth of the tumor from the epidermal granular layer to the deepest point of infiltration, or from the base of an ulcer to the maximum depth in case of ulceration. According to the National Comprehensive Cancer Network (NCCN) guidelines, safe surgical margins range from 0.5 to 1.0 CM for melanoma in situ and 2 CM for melanoma with a thickness of more than 2 mm. Surgical removal of the treatable is about 80% of the cases of CM [4,5]. Therefore, accurate measurement of the Breslow thickness before surgery is crucial for the primary surgical excision.

While contemporary dermatologic practice increasingly incorporates non-invasive imaging tools (e.g., dermoscopy, RCM, HFUS, line-field confocal OCT) as recommended by current international guidelines, histopathological examination from biopsy remains the reference standard for definitive diagnosis and Breslow thickness assessment in most clinical settings [6,7]. The common biopsy methods include excisional biopsy and partial biopsy (such as incision biopsy and scraping biopsy), with excisional biopsy being the preferred choice. However, excisional biopsy can be challenging for certain types of CMs, particularly those larger than 2 cm or located in specific areas like the face, palms, soles, ears, fingers, toes, or under the nails [8]. This led to the frequent use of partial biopsies.

Nonetheless, the biopsies cannot reflect the whole picture of the lesion. Therefore, the Breslow thickness of CM lesions cannot be accurately measured by biopsy. A meta-analysis revealed that biopsy samples often show high positivity rates for bottom margins, especially when the Breslow thickness exceeds 2 mm, with a 90% likelihood of a positive biopsy sample bottom margin [9,10]. Consequently, biopsies may underestimate the depth of tumor invasion, potentially resulting in incorrect initial surgical plans for up to 18% of cases, contrary to NCCN guidelines [11,12,13,14]. Some experimental and retrospective data suggest that surgical disruption of the tumor–lymphatic environment may theoretically influence melanoma progression; however, this remains investigational and is not definitively established in clinical practice [15,16]. Therefore, noninvasive methods with deep detection limits are essential for accurate measurement Breslow thickness of CM.

High-frequency ultrasound (HFUS ≥ 15 MHz) is a non-invasive method used for examining skin tumors [17]. It has significant clinical implications for preoperative diagnosis, staging, and treatment planning [18,19,20]. Previous studies have demonstrated the value of HFUS in the study of malignant skin tumors such as squamous cell carcinoma and basal cell carcinoma, as well as extramammary Paget’s disease. And proves the consistency of the HFUS image in extramammary Paget’s disease with pathological tissue [21,22]. HFUS can adjust the penetration of different frequencies to observe the base of CM lesions. This helps prevent underestimation of Breslow thickness and provides more precise information for treatment decisions [23,24].

Previous studies have reported a good correlation between HFUS-measured melanoma thickness and histologic Breslow depth, particularly in thicker lesions, but have not directly compared HFUS with partial biopsy [25,26]. Our work specifically compares HFUS with partial biopsy to determine whether HFUS can provide a more reliable preoperative thickness estimate.

## 2. Methods

This retrospective investigation was approved by the Ethics Committee of the University Hospital. Written informed consent from participants was not required in accordance with local/national guidelines. All procedures were performed according to the principles of the Declaration of Helsinki for Medical Research.

### 2.1. Patients

**Figure 1:**
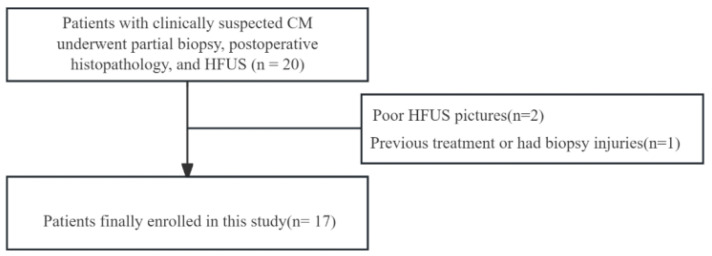
Flowchart for selection of the participants of the study. CM, cutaneous melanoma; HFUS, high-frequency ultrasound.

Databases were reviewed from February 2019 to October 2023 to identify eligible patients. Inclusion criteria were retrievable information regarding partial biopsy, postoperative histopathology, and ultrasound images of the CM. Patients who underwent previous treatment or had biopsy injuries and those with poor image quality were excluded from the study (Figure 1). The clinical features of the patients were collected, including age, sex, and lesion location.

### 2.2. Outcome and Measurement

During the study, the patient first underwent HFUS, followed by a partial biopsy, before surgery. The Breslow thicknesses obtained from both examinations were recorded. The Breslow thickness reported by postoperative histopathology was used as the gold standard to compare the two examination methods. Tumor T staging depended on the Breslow thickness of the CM and the presence of ulceration. Based on Breslow thickness, the T stage of the CM was evaluated to determine the surgical plan. The status of the surgical margins (negative or not) was documented. All images were digitally stored after the examination.

### 2.3. HFUS Examinations

All CM examinations were performed by an experienced radiologist (W-W Ren) with 5 years of experience with skin ultrasound, who was blinded to pathological biopsy results. All HFUS scans were performed according to established guidelines for diagnosing skin diseases [23].

HFUS was performed in two stages. Initially, a linear array transducer at a relatively low frequency (10–22 MHz; Esaote SpA, Genoa, Italy) was used to locate and measure the thickest part of each lesion. Then, the thickness of thin lesions was measured using a high-frequency transducer (22–38 MHz; KOLO, Suzhou, China). The protocol was as follows. First, before the HFUS examination, images of the lesion’s appearance were stored in a database. The radiologist assisted the patient in maintaining the proper body position to ensure complete exposure of the lesion. Then, a standard gel was applied to the transducer surface, and the transducer was placed vertically on the skin surface above the lesion. The amount of gel used ensured that there was no compression of the transducer and lesion surfaces. Finally, the transducer was manually held and moved in different directions along the skin surface until the thickest part of the lesion was located. On the two-dimensional image of HFUS, the CM appeared hypoechoic and irregular in shape. The radiologist measured and recorded the maximum thickness of the lesion based on clear images.

Considering the retrospective nature of this study, measures were taken to mitigate potential bias from the evaluator (W-W Ren) and their impact on image interpretation. Therefore, we enlisted two other experienced musculoskeletal radiologists, Q Wang, and D-D Shan, who had received training in the same evaluation standard for image analysis. The assessors evaluated the images without prior knowledge of the pathological findings, and the results of the two assessors were consistent.

### 2.4. Pathological Diagnosis

Although dermoscopy was routinely performed, it does not provide thickness assessment; therefore, the biopsy site was selected based mainly on the operator’s clinical judgment of the most suspicious or clinically thickest-appearing area. Partial biopsies were performed predominantly using incisional biopsy, with a minority using punch biopsy. A pathologist (N-H Wu) with over 10 years of experience diagnosing partial biopsy and postoperative specimens. He reported on the Breslow thickness, mitotic rate, melanoma subtype, ulcers, Clark levels, and immunohistochemistry (S-100 and HMB-45 staining). The thickness of nonulcerative lesions refers to the vertical distance from the top of the granular layer of the epidermis to the deepest part of the tumor infiltration. The thickness of the ulcerative lesion refers to the vertical distance from the base of the ulcer to the deepest part of the tumor infiltration. The Breslow thickness was measured in millimeters as a microscopic measurement and was accurate to 0.1 mm. The dermatopathologist assessing postoperative specimens was blinded to both HFUS measurements and partial biopsy thickness to avoid interpretive bias.

### 2.5. Statistical Analysis

Continuous variables of normal distribution are expressed as means standard deviations and those of nonnormal distribution as medians and interquartile ranges (IQR, 25th–75th percentile). The correlation between the Breslow thickness measured by partial biopsy, HFUS, and postoperative histopathology was assessed using Pearson’s correlation coefficient. Fisher’s test was used to establish associations between the collected variables and their effects on tumor T staging (underestimation or overestimation of T staging of the tumor) and surgical margins (expansion or inadequacy of surgical margins). Differences with *p* < 0.05 were considered to be statistically significant. All analyses were performed using SPSS version 25 (IBM Corporation, Armonk, NY, USA).

## 3. Results

### 3.1. Patient Characteristics

**Figure 2:**
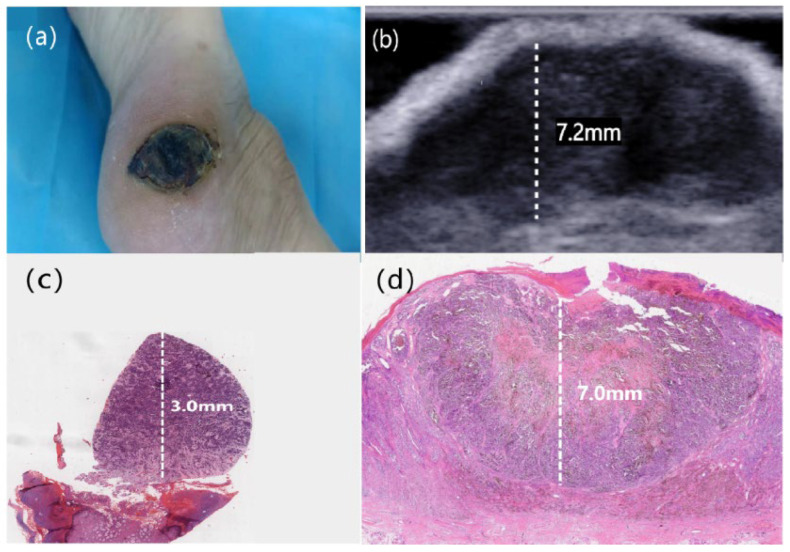
(**a**) A patient with right sole melanoma; (**b**) On the HFUS (Frequency: 22 MHz), the Breslow thickness was measured as 7.2 mm; (**c**) Partial biopsy was performed to obtain the pathological specimen, the Breslow thickness was 3 mm (original magnification ×40); (**d**) The pathological specimen obtained after surgical excision, the Breslow thickness was 7 mm (original magnification ×20).

Ultimately, data from 17 patients (12 [70.6%] female, 5 [29.4%] male; mean [±standard deviation (SD)] age, 68.6 ± 11.5 years [range, 43–89 years]) were retrospectively included in this study. A summary of the clinical data is presented in Table 1. Each patient contained one lesion. Thirteen patients (13/17 [76.5%]) had acral CM, 2 (2/17 [11.8%]) had lentigo CM, and 2 (2/17 [11.8%]) had nodular CM (Figure 2). The most common lesion sites were the feet (*n* = 14 [82.4%]), including the sole (*n* = 11) and toe (*n* = 3). In addition, 1 lesion occurred on the head and 2 lesions were on the finger. Thirteen (13/17 [76.5%]) patients had ulcer formation, and 7 (7/17 [41.2%]) patients had lymph node metastases.

### 3.2. Comparison of Measurements

The mean Breslow thicknesses measured using HFUS, partial biopsy, and postoperative histopathology were 3.5 ± 2.2 mm, 1.8 ± 1.1 mm, and 3.2 ± 2.1 mm, respectively. The agreement between the Breslow thickness measured using partial biopsy and postoperative histopathology was relatively poor (*r* = 0.76, *p* < 0.01). In contrast, the agreement between Breslow thickness measured by HFUS and postoperative histopathology was good (*r* = 0.96, *p* < 0.01) (Table 2). The maximum Breslow thickness measured using partial biopsy was 3.5 mm. However, the maximum Breslow thicknesses measured on postoperative histopathology and HFUS were 7.0 mm and 7.2 mm, respectively.

**Figure 3:**
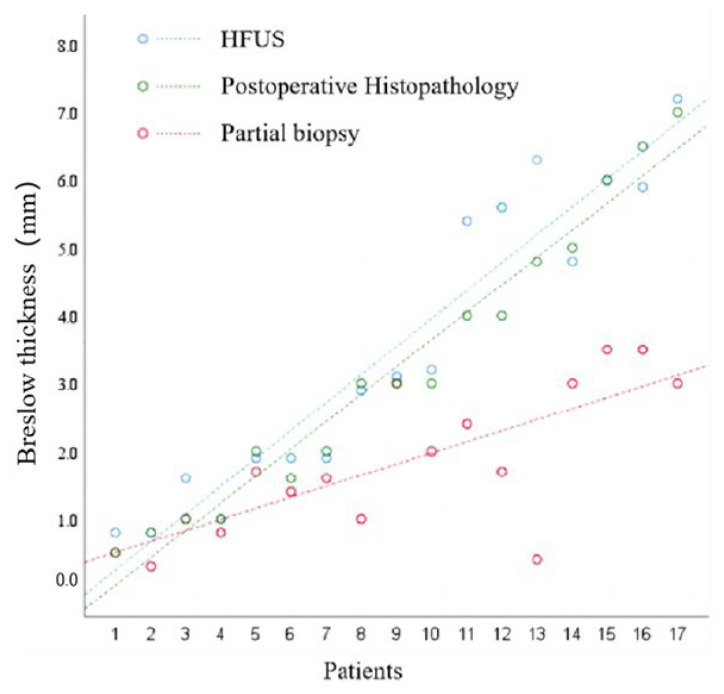
Consistency of the Breslow thickness measured by HFUS and Biopsy with Postoperative Histopathology. A scatter plot was developed with the patient number as the horizontal axis (sorted by Breslow thickness from lowest to highest) and the Breslow thickness measured by postoperative histopathology as the vertical axis while fitting a linear equation.

As shown in Figure 3, it is apparent that there is approximate parallel alignment between the HFUS and postoperative histopathology. However, the partial biopsy differs significantly from the two aforementioned alignments and is at a low level. This indicates that HFUS has a stronger correlation with postoperative histopathology than partial biopsy.

The CMs with different Breslow thicknesses ≤ 1.00 mm, 1.01–2.00 mm, 2.00–4.00 mm, and >4.00 mm were grouped based on the guidelines [27]. Of the 17 lesions subjected to partial biopsy, 9 lesions (9/17 [52.9%]) were in the same thickness range as the postoperative histopathology. However, for lesions with thicknesses 2.01–4.00 mm and those >4.00 mm, the Breslow thicknesses of 3 (3/17 [17.6%]) and 5 (5/17 [29.4%]) lesions were all underestimated. In contrast, for HFUS, there were 14 lesions (14/17 [82.4%]) in the same thickness range as postoperative histopathology. Only 1 (1/17 [5.9%]) lesion with thickness ≤ 1.00 mm, and 2 (2/17 [11.8%]) in the range of 2.01–4.00 mm were found to be overestimations of Breslow thickness (Table 3).

**Figure 4:**
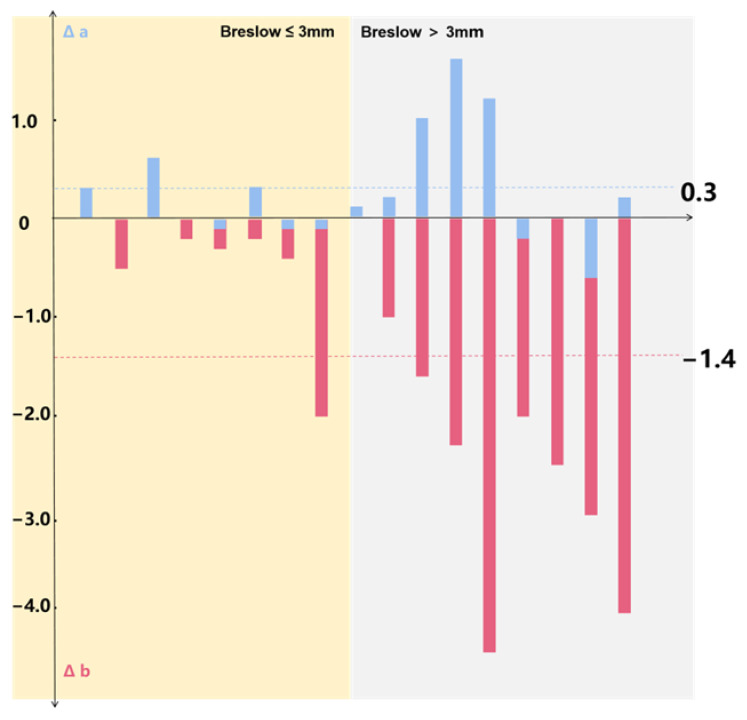
The result of subtraction between the Breslow thickness measured by HFUS, partial biopsy, and postoperative histopathology, respectively. Δa Breslow thickness measured by HFUS versus postoperative histopathological measurements. Δb Breslow thickness measured by partial biopsy versus postoperative histopathological measurements.

Comparison was made between the Breslow thicknesses measured by HFUS and partial biopsy with those measured by postoperative histopathology. It was found that the mean of the overestimation of Breslow thickness was 0.3 mm, while the mean underestimation was −1.4 mm. It became increasingly obvious that Breslow thickness was underestimated by partial biopsy as the actual thickness increased gradually (Figure 4).

**Figure 5:**
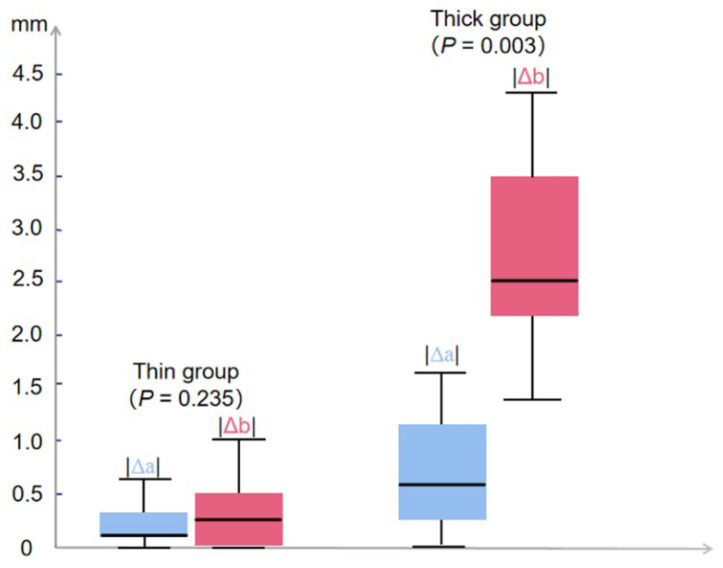
The difference between |Δa| and |Δb|, thin and thick group.

Taking the median Breslow thickness of 3 mm as the threshold, 17 patients were divided into two groups: thin group (Breslow thickness ≤ 3 mm), and thick group (Breslow thickness > 3 mm). Subsequently, we compared two groups of |Δa| (The absolute value of Δa) and |Δb| (The absolute value of Δb). The results showed that |Δa| was not statistically different from |Δb| in the thin group (*p* = 0.235). In contrast, in the thick group, |Δa| was statistically different from |Δb| (*p* = 0.003) (Figure 5).

The Breslow thicknesses measured in partial biopsy led to an underestimation of T staging in 8 patients (8/17 [47.1%]). According to the NCCN guidelines for CM, 4 out of 8 (50.0%) patients may have undergone surgery with insufficient peripheral surgical margins. Conversely, the Breslow thicknesses measured using HFUS led to a significant downward trend of underestimation (0/17 [0%], *p* = 0.003), but only a slight overestimation for 2 patients with T staging (2/17 [11.8%], *p* = 0.485). However, none of the overestimations changed the peripheral surgical margins (Table 4). Although a there was a downward trend in the rate of surgical margin change with HFUS was observed, it was not statistically significant compared to partial biopsy (*p* = 0.103).

## 4. Discussion

We conducted a retrospective study to compare partial biopsies and HFUS measurements of Breslow thickness in 17 patients with CM. Their consistency with postoperative histopathological results was analyzed. Our results revealed that, for measurements of Breslow thickness, HFUS demonstrated better agreement with postoperative histopathology than partial biopsy, especially for lesions thicker than 3 mm.

The results revealed that the Breslow thickness measured by HFUS was comparable with that measured by postoperative histopathology. However, there was a slight tendency to overestimate the mean measured by HFUS. This may be due to the presence of active lymphocytes near the bottom of the CM lesion, as HFUS is unable to differentiate between lymphocytes and tumor cells [28,29]. Therefore, inflammation may lead to a slight overestimation of CM thickness based on the current resolution of HFUS. Additionally, tissue specimens undergo dehydration and shrinkage after a series of treatments including complete excision, sectioning, fixation, and tumor staining [30]. However, HFUS measurements were taken on undehydrated lesions in vivo, which may also have resulted in slightly greater Breslow thicknesses.

After analyzing the data, we found that the correlation between Breslow thickness on both HFUS and partial biopsy decreased for thicker lesions (i.e., >3.0 mm) compared with postoperative histopathology. However, due to the limited sample size of this study, it was difficult to compare correlation coefficients. Interestingly, we observed that the Breslow thickness measurement of thicker lesions using the partial biopsy method seemed to be significantly inaccurate, with a trend of underestimation. This inaccuracy could be attributed to the “upper limitation” phenomenon observed in this study, where the measurement appeared to have an upper limit of approximately 3.5 mm. This phenomenon might be due to dermatologists trying to avoid excessive invasion, which leads to insufficient sampling. Theoretically, in addition to the upper sampling limitations, partial biopsies have significant uncertainty and do not accurately reflect the entire lesion. This is because biopsies are performed by dermatologists or pathologists who identify the biopsy site based on their experience. As a result, there are significant variations in the technique, tissue handling, and storage [31].

In addition, our data revealed that the HFUS-related overestimation of Breslow thickness did not affect the width of surgical margins. However, the partial biopsy-related underestimation led to insufficient surgical margins, which is consistent with previous studies [9,16,32]. According to NCCN guidelines [14], all CMs with a thickness of more than 2 mm are required for surgical margins of 2 cm. For such CMs, incorrect Breslow thickness does not affect the surgical margins. However, it may lead to inaccurate clinical staging, resulting in inadequate follow-up and treatment for certain patients. In this study, we found that Breslow thickness measured by partial biopsy decreased the T staging in 47.1% of CMs. While definitive T staging and follow-up decisions are based on the postoperative histopathologic Breslow thickness, preoperative underestimation by partial biopsy can still affect initial surgical management. An inaccurately low thickness estimate may lead to suboptimal excision margins or omission of a sentinel lymph node biopsy, potentially necessitating secondary procedures. Thus, the clinical impact of biopsy underestimation lies primarily in early surgical planning rather than long-term staging. HFUS, by providing a more accurate preoperative thickness measurement, may help mitigate this limitation. In this study, HFUS demonstrated strong accuracy in assessing Breslow thickness of the primary melanoma, supporting its value as a noninvasive tool for preoperative characterization of the primary lesion.

Of course, this research also has several limitations. First, the study design was retrospective and conducted at a single center with a small sample size. Second, as HFUS is an operator-dependent technology, our study was initially examined and read by one radiologis and then read a second time by two other radiologists. Despite this, there were still inevitable problems of consistency, both between different operators and within the same operator. It is necessary to establish a standardized method of measuring HFUS in the preoperative evaluation of CM. Because biopsy site selection relied on operator judgment, suboptimal targeting of the true maximal-thickness area may have contributed to the underestimation of Breslow thickness observed in partial biopsies. Our next course of action will involve conducting a large-sample, prospective, controlled study across multiple centers to validate our findings.

To sum up, HFUS can accurately assess the Breslow thickness before surgery. Compared to partial biopsy, HFUS measurements show a better correlation with postoperative histopathology, especially for thick CMs (>3.0 mm). Although HFUS slightly overestimated the Breslow thickness and T staging for CMs, there was no statistically significant difference.

## Figures and Tables

**Table 1:** Basic patient characteristics and clinical data on CM.

Patient	Sex	Age, y	Location	Ulceration	Lymph Node Metastasis
1	Male	65	Finger	Present	No
2	Female	74	Head	Present	No
3	Male	69	Sole	Absent	No
4	Female	50	Finger	Present	No
5	Female	87	Sole	Absent	No
6	Female	66	Sole	Present	Yes
7	Female	63	Sole	Present	No
8	Female	68	Toe	Present	Yes
9	Female	67	Sole	Present	Yes
10	Female	62	Sole	Present	Yes
11	Female	64	Sole	Present	No
12	Male	76	Toe	Absent	No
13	Male	43	Sole	Present	No
14	Male	89	Toe	Present	Yes
15	Female	81	Sole	Absent	No
16	Female	73	Sole	Present	Yes
17	Female	69	Sole	Present	Yes

**Table 2:** The comparison of Breslow Thickness among HFUS, Partial Biopsies, and Postoperative Histopathology.

Breslow Thickness (mm)	HFUS	Partial Biopsy	Postoperative Histopathology
Mean	3.5	1.8	3.2
SD	2.2	1.1	2.1
Median	3.1	1.7	3.0
Range	0.8–7.2	0.5–3.5	0.5–7.0
Pearson coefficient	0.96 (*p* < 0.01)	0.76 (*p* < 0.01)	-

**Table 3:** Consistency of the CM’s Breslow Thickness measured by HFUS and Partial Biopsy with Postoperative Histopathology.

Postoperative Histopathology	HFUS				Partial Biopsy			
	≤1.00 mm	1.01–2.00 mm	2.01–4.00 mm	>4.00 mm	≤1.00 mm	1.01–2.00 mm	2.01–4.00 mm	>4.00 mm
≤1.00 mm	3	1 *	0	0	4	0	0	0
1.01–2.00 mm	0	3	0	0	0	3	0	0
2.01–4.00 mm	0	0	3	2 *	1 #	2 #	2	0
>4.00 mm	0	0	0	5	1 #	0	4 #	0

* Overestimation of Breslow thickness measurements by HFUS. # Underestimation of Breslow thickness measurements by partial biopsy.

**Table 4:** The change of the T staging and the surgical peripheral margins between HFUS and partial biopsy.

Variables	HFUS	Partial Biopsy	*p*-Value
T staging Underestimation	0/17 (0%)	8/17 (47.1%)	0.003 *
T staging Overestimation	2/17 (11.8%)	0/17 (0%)	0.485
Surgical margins changing	0/17 (0%)	4/17 (23.5%)	0.103

*: Indicates a significant difference in the underestimation of T stage.

## Data Availability

Due to information that may compromise the privacy of study participants, the data supporting the findings of this study is not publicly available, but is available from the corresponding author [D Shan and C Qin]. The recipient should provide reasonable requirements and contact information (including email address).
